# Progress in the management and outcome of small-cell lung cancer in a French region from 1981 to 1994

**DOI:** 10.1054/bjoc.2001.1955

**Published:** 2001-09

**Authors:** M P Lebitasy, G Hédelin, A Purohit, L Moreau, F Klinzig, E Quoix

**Affiliations:** Service de Pneumologie Lyautey, CHU de Strasbourg, 1 rue des Canonniers, Strasbourg, 67100; Laboratoire d'Épidémiologie et de Santé Publique, Université Louis Pasteur, Faculté de Médecine, 4 rue Kirschleger, Strasbourg, 67085, France

**Keywords:** small-cell lung cancer, survival, management, chemotherapy, prognosis

## Abstract

Recent analyses of series of small-cell lung cancer (SCLC) patients included in clinical trials have shown improved survival over time, but it has been impossible to determine whether this was due to selection biases, stage migration, or true therapeutic improvement. To determine if there has been a true improvement of survival over time, we reviewed the medical records of all consecutive patients diagnosed with SCLC between 1981 and 1994 in the Bas-Rhin in France. Among the 787 patients (median age 63), there was no significant period effect for sex, age, or stage. Staging work-ups became increasingly thorough (significant period effect). The mean number of investigations and of tumour sites detected correlated significantly. The chemotherapy rate increased (from 76.4% in 1981–1983 to 91.7% in 1993–1994, *P* = 10^−5^) and mediastinal irradiation decreased (to roughly 25% of patients after 1983). Median survival time increased for the overall population from 6.6 months in 1981–1983 to 11.3 months in 1993–1994 (*P* = 10^−5^), for patients with limited disease (LD) from 9.2 (*P* = 0.002) months to 14.0 months, and for those with extensive (ED) disease from 3.5 months to 9.6 months (*P* = 10^−5^). Significant independent prognostic factors were disease extent, clinical trial participation, period, type of chemotherapy, and mediastinal irradiation in LD. Survival time has truly improved as ‘state of the art' management of SCLC has changed. © 2001 Cancer Research Campaignhttp://www.bjcancer.com


*British Journal of Cancer* (2001) **85**(6), 808–815
© 2001 Cancer Research Campaign
doi: 10.1054/ bjoc.2001.1955, available online at http://www.idealibrary.com on IDEAL

http://www.bjcancer.com

### Progress in the management and outcome of small-cell lung cancer in a French region from 1981 to 1994

**MP Lebitasy[1], G Hédelin[2], A Purohit[1], L Moreau[1], F Klinzig[1] and E Quoix[1]**

[1]Service de Pneumologie Lyautey, CHU de Strasbourg, 1 rue des Canonniers, 67100 Strasbourg; and [2] Laboratoire d'Épidémiologie et de Santé Publique, Université Louis Pasteur, Faculté de Médecine, 4 rue Kirschleger, 67085 Strasbourg, France

**Summary** Recent analyses of series of small-cell lung cancer (SCLC) patients included in clinical trials have shown improved survival over time, but it has been impossible to determine whether this was due to selection biases, stage migration, or true therapeutic improvement. To determine if there has been a true improvement of survival over time, we reviewed the medical records of all consecutive patients diagnosed with SCLC between 1981 and 1994 in the Bas-Rhin in France. Among the 787 patients (median age 63), there was no significant period effect for sex, age, or stage. Staging work-ups became increasingly thorough (significant period effect). The mean number of investigations and of tumour sites detected correlated significantly. The chemotherapy rate increased (from 76.4% in 1981–1983 to 91.7% in 1993–1994, $P = 10^{-5}$) and mediastinal irradiation decreased (to roughly 25% of patients after 1983). Median survival time increased for the overall population from 6.6 months in 1981–1983 to 11.3 months in 1993–1994 ($P = 10^{-5}$), for patients with limited disease (LD) from 9.2 ($P = 0.002$) months to 14.0 months, and for those with extensive (ED) disease from 3.5 months to 9.6 months ($P = 10^{-5}$). Significant independent prognostic factors were disease extent, clinical trial participation, period, type of chemotherapy, and mediastinal irradiation in LD. Survival time has truly improved as 'state of the art' management of SCLC has changed. © 2001 Cancer Research Campaign http://www.bjcancer.com

**Keywords**: small-cell lung cancer; survival; management; chemotherapy; prognosis

Lung cancer is the most frequent cause of cancer death among men in all developed countries (Parkin et al, 1993) and, in most of the United States, for women as well (Silverberg, 1998). In Europe, lung cancer is the third leading cause of cancer death in women (Black et al, 1997); in France, it is fourth (Black et al, 1997). Small-cell lung cancer (SCLC) accounts for 20–25% of all lung cancers (Carter and Eggleston, 1980; Janssen-Heijnen et al, 1995; WHO, 1998). Its prognosis remains poor, with a median survival time of about 12–16 months in LD and 7–11 months in ED (Albain et al, 1990).

Management of SCLC has changed substantially over time. During the 1960s, surgery was found to be useless (Fox and Scadding, 1973). As mediastinal irradiation became the most frequently used treatment, disease was staged by the VALSG (Veterans' Administration Lung Study Group) two-category classification: the LD stage, in which the tumour and its connections can be encompassed by a single radiotherapy port and the ED stage, in which the tumour extends beyond the LD definition (Zelen, 1973). Since 1969, chemotherapy became the cornerstone of SCLC therapy (Green et al, 1969). The most frequently used drug combinations were first CAV (cyclophosphamide, adriamycin and vincristine) (Livingston et al, 1978; Aisner et al, 1983) and then etoposide with cisplatin, which is today the standard treatment (Evans et al, 1985; Fukuoka et al, 1991; Roth et al, 1992). Two meta-analyses of 13 randomized trials comparing chemotherapy alone with a combination of chemotherapy and radiotherapy for LD patients concluded that combined treatment in LD provided a small but definite survival benefit (Pignon et al, 1992; Warde and Payne, 1992). In ED, chemotherapy alone is the usual treatment. Nonetheless, despite extreme initial sensitivity to chemotherapy, most patients with SCLC relapse and become resistant to chemotherapy (Giaccone et al, 1988).

Improved survival rates over time have been reported in analyses of series of patients included in consecutive therapeutic trials over periods of several years (Spiegelman et al, 1989; Albain et al, 1990; Chute et al, 1999). This improvement in survival over time for patients in clinical trials may be due to many factors, including stage migration (Feinstein et al, 1985) and selection and lead-time biases (Antman et al, 1985; Quoix et al, 1986). The possible role of improved therapy per se cannot therefore be assessed in such studies. Few population-based studies have been performed demonstrating, in the recent series, also improvement in survival (Janssen-Heijnen et al, 1998a, 1998b; Engeland et al, 1998). We decided to conduct a retrospective study of the management of all consecutively diagnosed patients with SCLC in the Bas-Rhin (a French department, an administrative subdivision) over a 14-year period to analyse their outcome and assess trends in management and survival.

*Received 11 September 2000*
*Revised 17 May 2001*
*Accepted 1 June 2001*

*Correspondence to:* E Quoix

### PATIENTS AND METHODS

### Patients

We reviewed the medical records of all 967 patients diagnosed with SCLC between 1 January 1981 and 31 December 1994 in the Bas-Rhin, which had between 900 000 and 1 000 000 inhabitants during this period (INSEE, 1987). We obtained the list of patients from the Bas-Rhin population-based cancer-registry.

Each chart included the following data: age, sex, date of diagnosis, investigations performed for diagnosis and staging purposes, disease extent according to the VALSG classification (Zelen, 1973) and serum lactate dehydrogenase (LDH) levels. If the chart did not report disease extent at diagnosis, we assigned the stage retrospectively if at least two extra-thoratic procedures were performed or, of course, when a metastatic site was found (the patient was then classified as ED regardless of the number of procedures). Otherwise, the stage was classified as undetermined.

We recorded the first-line treatment received by the patient: surgery, chemotherapy and/or mediastinal irradiation, prophylactic cranial irradiation. The drugs used, the number of cycles administered, and the total dose of irradiation delivered (Grays) were noted. Participation in any clinical trial was also recorded.

Survival was calculated from the date of pathological diagnosis to the end point date which was 31 December 1998.

### Statistical methods

### *Descriptive analysis*

All variables recorded were divided into five periods; four covered 3 years and the last period, only 2 (1993–1994).

Differences between proportions were evaluated with the Pearson $\chi^2$ test or Fisher's exact test (Armitage, 1971).

Differences between the means of continuous variables were evaluated with Student's *t*-test or, if the comparison involved more than two groups, one-way analysis of variance (Armitage, 1971).

Correlation between continuous variables was assessed with the correlation test (Schwartz, 1996).

In the descriptive analysis, if both period effect and linear trend were significant, only the latter's *P*-value is indicated, because it necessarily includes the period effect. When there was a period effect without a significant linear trend, both significance values are indicated.

All continuous variables were categorized before analysis. Age was categorized in three classes (less or equal to 55, from 56 to 70 and over 70). LDH dosage was categorized as normal or elevated.

The BMDP package from the University of California, Los Angeles, CA (BMDP, 1981) and STATXACT of Cytel Software Corporation, Cambridge (STATXACT, 1995) were used to process the data.

### *Relative survival*

RELSURV software (2.0c) (Hédelin, 1995) was used to perform univariate and multivariate analysis of survival with relative survival rates. Considering relative survival enabled us to eliminate the effect of improvement in general population survival during the long period (14 years) covered by the study as well as the effect of age-related mortality.

Several pre-therapeutic and therapeutic variables were studied: sex, age, disease extent, inclusion in a clinical trial, LDH, diagnosis period, chemotherapy and mediastinal irradiation.

Combined chemotherapies were categorized in five groups: cisplatin and etoposide solely, cisplatin with other drug(s) except etoposide, etoposide with other drug(s) except cisplatin, neither cisplatin nor etoposide, cisplatin and etoposide with other drug(s).

### RESULTS

### Descriptive analysis

Of the 967 patients with SCLC listed in the cancer registry of the Bas-Rhin during this period, 180 were excluded, for two reasons: ineligibility due to wrong histological diagnosis (25 patients), absence of or insufficient medical records (155 patients). This study thus included 787 patients (83.4% of all eligible patients).

The sex ratio did not differ significantly between the 155 eligible patients excluded from this study and the 787 included: 133 (85.8%) and 696 (88.4%) men, respectively. The median age of the excluded patients was 64, and of the included patients, 63 ($P = 0.05$). Among the excluded patients, 67% were diagnosed before 1988, compared with 46.8% of those included ($P = 10^{-5}$).

Nineteen (2.4%) of the included patients were still alive at the end point: 766 (97.3%) had died and 2 patients (0.3%) were lost to follow-up.

Table 1 summarizes the patients' characteristics and the temporal trends and Table 2 reports the procedures performed (bronchofiberoscopy and imaging) over time.

Finally, 248 patients (31.5%) were classified as LD at diagnosis and 446 patients (56.7%) as ED. Inadequate testing prevented

**Table 1** Characteristics of study population

| Period | 1981–83 | | 1984–86 | | 1987–89 | | 1990–92 | | 1993–94 | | *P* |
|---|---|---|---|---|---|---|---|---|---|---|---|
| | No. | % | No. | % | No. | % | No. | % | No. | % | |
| Sex | | | | | | | | | | | |
| Male | 127 | 90.7 | 149 | 87.1 | 148 | 90.8 | 168 | 87.5 | 104 | 86.0 | 0.60 |
| Female | 13 | 9.3 | 22 | 12.9 | 15 | 9.2 | 24 | 12.5 | 17 | 14.0 | |
| Age (years) | | | | | | | | | | | |
| <=55 | 44 | 31.4 | 39 | 22.8 | 43 | 26.4 | 56 | 29.2 | 23 | 19.0 | |
| 56–70 | 64 | 45.7 | 84 | 49.1 | 83 | 50.9 | 98 | 51.0 | 60 | 49.6 | 0.19 |
| >70 | 32 | 22.9 | 48 | 28.1 | 37 | 22.7 | 38 | 19.8 | 38 | 31.4 | |
| 'on' prot | 6 | 4.4 | 11 | 6.7 | 67 | 41.9 | 32 | 17.0 | 16 | 13.7 | 0.002 |
| Dis Ext* | | | | | | | | | | | |
| LD | 43 | 39.4 | 47 | 33.3 | 49 | 33.3 | 66 | 36.1 | 43 | 37.7 | 0.81 |
| ED | 66 | 60.6 | 94 | 66.7 | 98 | 66.7 | 117 | 63.9 | 71 | 62.3 | |
| Stage ND | 31 | 22.1 | 30 | 17.5 | 16 | 9.8 | 9 | 4.7 | 7 | 5.8 | $10^{-5}$ |

*Proportion of defined stages; Dis Ext: disease extent; Stage ND: not determined; 'on' prot: 'on' protocol.

© 2001 Cancer Research Campaign

**Table 2** Period effect for investigations performed

| Period | 1981–83 | | 1984–86 | | 1987–89 | | 1990–92 | | 1993–94 | | *P* |
|---|---|---|---|---|---|---|---|---|---|---|---|
| | No. | % | No. | % | No. | % | No. | % | No. | % | |
| Fiberoscopy | 136 | 97.8 | 164 | 95.9 | 160 | 98.2 | 186 | 97.4 | 117 | 96.7 | 0.75 |
| Thorax CT | 7 | 5.1 | 44 | 27.2 | 92 | 56.4 | 160 | 85.6 | 110 | 91.7 | $10^{-5}$ |
| Abdom US* | 121 | 88.3 | 163 | 97.0 | 159 | 98.1 | 179 | 96.2 | 112 | 93.3 | 0.0007 |
| Adrenal CT | 2 | 1.4 | 22 | 13.0 | 70 | 44.6 | 123 | 66.5 | 78 | 65.0 | $10^{-5}$ |
| Brain CT | 7 | 5.2 | 45 | 27.3 | 103 | 64.8 | 158 | 85.4 | 102 | 87.2 | $10^{-5}$ |
| Bone scan | 83 | 61.0 | 110 | 65.9 | 105 | 66.0 | 116 | 66.3 | 72 | 67.3 | 0.84 |
| BM Biopsy* | 33 | 25.2 | 64 | 39.0 | 74 | 46.5 | 69 | 39.4 | 33 | 29.5 | 0.002 |

*Linear trend not significant; Thorax CT: Thoracic CT- scan; Abdom US*: Abdominal ultrasound, $P = 0.12$ for linear trend; Adrenal CT: Adrenal CT-scan; Brain CT: Brain CT-scan; BM Biopsy*: Bone marrow biopsy, $P = 0.40$ for linear trend.

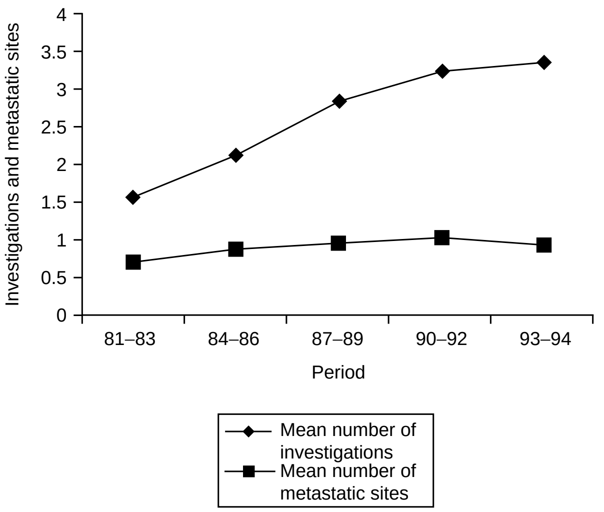

**Figure 1** Correlation between investigations and metastatic sites ($r = 0.184$; $P < 0.001$)

stage determination for 93 patients (11.8%). The proportion of patients whose stage was undetermined decreased dramatically over time (Table 1).

The mean number of metastatic sites detected increased slightly over time without any significant period effect ($P = 0.09$). There was a significant correlation between the mean number of imaging procedures and the mean number of sites detected with tumoral involvement (Figure 1).

Most of the patients were treated in the University Hospital of Strasbourg (80.5%), but 17.2% were treated in community hospitals and 2.3% in private clinics. There was a period effect for each type of treatment: chemotherapy, mediastinal radiotherapy, prophylactic cranial irradiation and surgery (Table 3).

One hundred twenty-six patients (16%) did not receive any chemotherapy. The median age of these patients was 73, compared with 61 for those who did ($P = 10^{-5}$). In the no-chemotherapy group, there was no significant period effect for sex and age distribution. The proportion of patients who had no chemotherapy decreased significantly over time: from 23.6% during 1981–83 to 8.3% during 1993–94 ($P = 10^{-5}$).

There was a period effect for most drugs (Table 4). The use of cisplatin and etoposide increased over time, and the use of vincristine, lomustine and methotrexate decreased. Anthracyclines were given to roughly 70% of patients throughout the entire period. The mean number of cycles administered varied over time ($P = 10^{-4}$) without a linear trend ($P = 0.19$), from 5.1 in 1981–83 to 5.3 in 1993–94, with a peak of 6.2 in 1984–86.

After the first period (1981–1983), the proportion of patients receiving mediastinal irradiation decreased to about 24–25%. The number of patients undergoing prophylactic cranial irradiation also decreased substantially after the first period. Finally, the rate of surgical resection remained minimal throughout the period.

The proportion of patients included in clinical trials increased irregularly over time, from 4.4% of all patients in 1981–83 to 13.7% in 1993–94, with a peak at 41.9% in 1987–89 ($P = 0.002$). All these patients were treated in the University Hospital.

### Univariate analysis of survival

Sixteen patients died before diagnosis and are excluded from further analysis of survival.

The overall median survival time increased over time, from 6.6 months in 1981–83 to 11.3 months in 1993–94 ($P = 10^{-5}$). This improvement was observed for both LD and ED patients (Figure 2): for LD patients, median survival increased from 9.2 months in 1981–83 to 14.0 months in 1993–94 ($P = 0.002$) and for ED

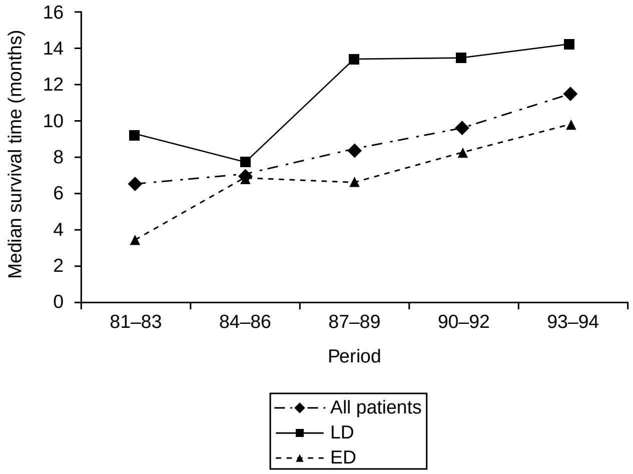

**Figure 2** Period effect for median survival time. All patients: $P = 10^{-5}$.LD: $P = 0.002$. ED: $P = 10^{-5}$. ($P$ = significance for linear trend)

 *© 2001 Cancer Research Campaign*

**Table 3** Period effect for therapy

| Period | 1981–83 | | 1984–86 | | 1987–89 | | 1990–92 | | 1993–94 | | *P* |
|---|---|---|---|---|---|---|---|---|---|---|---|
| | No. | % | No. | % | No. | % | No. | % | No. | % | |
| CT | | | | | | | | | | | |
| All | 107 | 76.4 | 133 | 77.8 | 138 | 84.7 | 172 | 89.6 | 111 | 91.7 | $10^{-5}$ |
| LD | 37 | 86.0 | 39 | 83.0 | 46 | 93.9 | 60 | 90.9 | 40 | 93.0 | 0.36 |
| ED | 45 | 68.2 | 71 | 75.5 | 83 | 84.7 | 109 | 93.2 | 66 | 93.0 | $10^{-5}$ |
| Med RT | | | | | | | | | | | |
| All | 74 | 52.9 | 49 | 29.0 | 39 | 24.1 | 47 | 24.5 | 30 | 24.8 | $10^{-5}$ |
| LD* | 34 | 79.1 | 20 | 44.4 | 23 | 46.9 | 40 | 60.6 | 25 | 58.1 | 0.008 |
| ED | 24 | 36.4 | 19 | 20.2 | 7 | 7.1 | 5 | 4.3 | 2 | 2.8 | $10^{-5}$ |
| CT + Med RT | | | | | | | | | | | |
| All | 58 | 41.4 | 34 | 19.9 | 32 | 19.6 | 43 | 22.4 | 29 | 24.0 | 0.006 |
| LD* | 29 | 67.4 | 14 | 29.8 | 22 | 44.9 | 37 | 56.1 | 24 | 55.8 | 0.005 |
| ED | 15 | 22.7 | 12 | 12.8 | 5 | 5.1 | 5 | 4.3 | 2 | 2.8 | $10^{-5}$ |
| PCI | | | | | | | | | | | |
| All | 25 | 17.9 | 8 | 4.7 | 5 | 3.1 | 3 | 1.6 | 6 | 5.0 | $10^{-5}$ |
| LD | 17 | 39.5 | 4 | 8.7 | 4 | 8.2 | 3 | 4.6 | 5 | 11.6 | $10^{-4}$ |
| ED | 8 | 12.1 | 3 | 3.2 | 1 | 1.0 | 0 | 0.0 | 0 | 0.0 | $10^{-5}$ |
| Surgery | | | | | | | | | | | |
| All | 4 | 2.9 | 7 | 4.1 | 8 | 4.9 | 1 | 0.5 | 1 | 0.8 | 0.05 |
| LD | 2 | 4.7 | 4 | 8.7 | 6 | 12.5 | 0 | 0.0 | 0 | 0.0 | 0.05 |
| ED | 1 | 1.5 | 2 | 2.1 | 0 | 0.0 | 1 | 0.9 | 1 | 1.4 | 0.70 |

*Linear trend not significant; CT: Chemotherapy; Med RT: Mediastino-tumoral radiotherapy, for LD $P = 0.34$ for linear trend; CT + Med RT: Chemotherapy plus mediastino-tumoral radiotherapy, for LD $P = 0.75$ for linear trend; PCI: Prophylactic cranial irradiation; For each treatment type, the number of LD + ED patients is inferior to the number of all patients in which patients with not determined stage are also present.

**Table 4** Period effect for drugs used

| Period | 1981–83 | | 1984–86 | | 1987–89 | | 1990–92 | | 1993–94 | | *P* |
|---|---|---|---|---|---|---|---|---|---|---|---|
| | No. | % | No. | % | No. | % | No. | % | No. | % | |
| Anthr | 77 | 72.0 | 94 | 70.7 | 99 | 72.3 | 103 | 59.9 | 77 | 69.4 | 0.10 |
| VCR | 85 | 79.4 | 83 | 62.4 | 43 | 31.4 | 21 | 12.2 | 21 | 18.9 | $10^{-5}$ |
| Cyclifo | 89 | 83.2 | 110 | 82.7 | 81 | 59.1 | 39 | 22.7 | 72 | 64.9 | $10^{-5}$ |
| VP16 | 22 | 20.6 | 93 | 69.9 | 93 | 67.9 | 149 | 86.6 | 95 | 85.6 | $10^{-5}$ |
| CDDP | 13 | 12.1 | 76 | 57.1 | 48 | 35.0 | 126 | 73.3 | 85 | 76.6 | $10^{-5}$ |
| Carbo* | 0 | 0.0 | 1 | 0.8 | 18 | 13.1 | 2 | 1.2 | 4 | 3.6 | $10^{-5}$ |
| Lomus | 61 | 57.0 | 30 | 22.6 | 18 | 13.1 | 13 | 7.6 | 6 | 5.4 | $10^{-5}$ |
| MTX | 76 | 71.0 | 34 | 25.6 | 16 | 11.7 | 16 | 9.3 | 6 | 5.4 | $10^{-5}$ |
| Other | 21 | 19.6 | 10 | 7.5 | 9 | 6.6 | 7 | 4.1 | 3 | 2.7 | $10^{-5}$ |

* Linear trend not significant; Anthr = Anthracyclines = Doxorubicine or Epirubicine; Cyclifo = Cyclophosphamide or Ifosfamide; VCR = Vincristine; VP16 = Etoposide; CDDP = Cisplatin; Carbo* = Carboplatine, $P = 0.27$ for linear trend; Lomus = Lomustine; MTX = Methotrexate.

patients from 3.5 months to 9.6 months ($P = 10^{-5}$). This period effect on survival was also observed and significant for patients included in clinical trials and for those not included.

The 1-year probability of survival increased from 24.2% in 1981–83 to 47.3% in 1993–94 for all patients ($P = 0.002$); the 2-year probability of survival also increased from 7.9% in 1981–83 to 17.1% in 1993–94 ($P = 0.03$). For LD and ED patients, respectively, the 1-year probability of survival increased from 42.2% and 12.1% in 1981–83 to 69.5% and 39.2% in 1993–94 ($P = 10^{-6}$ and 0.0003); the 2-year probability from 21.2% and 1.4% in 1981–83 to 32.3% and 9.3% in 1993–94 ($P = 10^{-6}$ and 0.006).

One hundred and eight patients (14%) died within a month of diagnosis: 51 (47.2%) received no treatment. The percentage of early deaths did not vary over time.

### Multivariate analysis of survival

Age, disease extent, inclusion in clinical trial, period, chemotherapy and mediastinal irradiation were studied in a forward stepwise fashion. LDH was omitted because of the numerous missing values (44.8%).

Two multivariate analyses were performed:

- First, an analysis with the pre-therapeutic variables: disease extent, inclusion in clinical trials, and period are independent prognostic factors after adjustment for other factors (Table 5).
- The next analysis took into account not only pre-therapeutic but also therapeutic factors (Table 6). Patients treated with cisplatin plus etoposide plus other drug(s) did better although not significantly. Mediastinal radiation

*© 2001 Cancer Research Campaign*

**Table 5** Multivariate analysis for pre-therapeutic variables

| Variable | Level | Hazard ratio | CI |
|---|---|---|---|
| Age (years) | < = 55 | 1 | |
| | 55–70 | 1.27 | 0.92–1.74 |
| | >70 | 1.51 | 0.96–2.40 |
| Disease extent* | LD | 1 | |
| | ED | 2.04 | 1.68–2.47 |
| Inclusion in trial | No | 1 | |
| | Yes | 0.60 | 0.47–0.76 |
| Period | 1981–83 | 1 | |
| | 1984–86 | 0.81 | 0.47–1.38 |
| | 1987–89 | 1.06 | 0.64–1.75 |
| | 1990–92 | 0.75 | 0.46–1.23 |
| | 1993–94 | 0.49 | 0.27–0.87 |

CI: Confidence interval; * The 93 patients with undetermined stage are excluded from the analysis.

**Table 6** Multivariate analysis for pre-therapeutic and therapeutic variables

| Variable | Level | Hazard ratio | CI |
|---|---|---|---|
| Age | < 55 | 1 | |
| | 56–70 | 1.19 | 0.9–1.59 |
| | >70 | 1.08 | 0.68–1.71 |
| Disease extent7 | LD | 1 | |
| | ED | 1.34 | 0.94–1.93 |
| Inclusion in trial | No | 1 | |
| | Yes | 0.72 | 0.56–0.92 |
| Period | 1981–83 | 1 | |
| | 1984–86 | 0.69 | 0.33–1.42 |
| | 1987–89 | 0.89 | 0.43–1.86 |
| | 1990–92 | 0.67 | 0.28–1.57 |
| | 1993–94 | 0.49 | 0.22–1.09 |
| Chemotherapy | Chemo 1 | 1 | |
| | Chemo 2 | 0.75 | 0.40–1.40 |
| | Chemo 3 | 0.82 | 0.39–1.71 |
| | Chemo 4 | 0.68 | 0.32–1.44 |
| | Chemo 5 | 0.67 | 0.40–1.12 |
| Radiotherapy | | | |
| LD | No | 1 | |
| | Yes | 0.52 | 0.31–0.89 |
| ED | No | 1 | |
| | Yes | 0.85 | 0.57–1.25 |

CI: Confidence interval; Chemo 1 = (Etoposide + Cisplatin) alone; Chemo 2 = Etoposide + other drug(s) (except Cisplatin); Chemo 3 = Cisplatin + other drug(s) (except Etoposide); Chemo 4 = neither Etoposide nor Cisplatin; Chemo 5 = Etoposide + Cisplatin + other drug(s).

had a significant favourable impact on survival in the LD stage.

### DISCUSSION

The distribution by sex, age and disease extent did not differ significantly over time among the patients diagnosed with SCLC in the Bas-Rhin from 1 January 1981 through 31 December 1994.

Although the sex ratio in our study decreased from 9.8:1 during the first period to 6.1:1 during the last period; this reduction was not significant. In a population-based study of SCLC performed in the Netherlands, the male/female ratio appeared to decrease from 14:1 in 1975–1979 to 4:1 in 1990–1994 (Janssen-Heijnen et al, 1998a). It is well known that the sex ratio is lower in Northern Europe than in the countries of Southern Europe (Black et al, 1997). The absence of a period effect on the sex ratio in our study is consistent with the late adoption of cigarette smoking by French women (European Bureau For Action on Smoking Prevention, 1990).

The median age in our series is 63, slightly lower than the 65 found in a Dutch population-based-analysis of SCLC patients diagnosed between 1975 and 1994 (Janssen-Heijnen et al, 1998a). For patients in clinical trials in our study population (16.7% of all patients), the median age was 59 years and the proportion of patients older than 60 years was 45.5%. Comparisons of age distribution in population-based series and in series of patients included in clinical trials are not appropriate because most trials have an upper age limit as an inclusion criteria. The median age of a population-based series may also reflect health policies and the country's socio-economic status. The percentage of elderly patients with SCLC may be under-estimated if elderly patients with lung tumour masses do not undergo diagnostic procedures at rates similar to younger patients.

In our population, 31.5% of the patients with known stage were diagnosed with LD, and this proportion did not vary over time. The proportion of patients whose disease stage was undetermined decreased over time (from 22.1% to 5.8%). A survey of lung cancer in the US also observed a dramatic decrease in the percentage of unstaged cases from 1985 to 1995 (Fry et al, 1999). In that study, as in ours, this change did not modify the distribution of disease stages.

The mean number of metastatic sites detected increased over time, together with the number of procedures performed for staging purposes. The increase in observed metastases probably also reflects improvements in imaging quality. This is consistent with other findings (Griffin et al, 1984; Spiegelman et al, 1989; Dearing et al, 1990).

We found that survival increased over time for the population as a whole and for each category (LD, ED, included or not in clinical trials). Retrospective studies of patients with SCLC in consecutive clinical trials (Spiegelman et al, 1989; Albain et al, 1990; Chute et al, 1999) have also observed improved survival. Many factors may account for the improvement in this group; they include better staging because of more and better staging procedures. The LD group may therefore be less 'contaminated' by small metastases undetectable with 'older' procedures; simultaneously the ED group may now include not only patients with massive metastatic disease but also some patients with small metastases detected with 'new' procedures (Feinstein et al, 1985). This stage migration, known as the 'Will Rogers phenomenon', results in an improved survival for LD and ED patients, without improvement of the overall survival. The improved survival of patients in consecutive clinical trials may therefore be explained simply by the Will Rogers phenomenon (Spiegelman et al, 1989; Albain et al, 1990; Chute et al, 1999) contrarily to our study.

In clinical trials, patient selection, through restrictive inclusion criteria, may also affect the improved outcome of included patients (Antman et al, 1985; Quoix et al, 1986). Again, this does not explain the global improved survival in our series but may have

 *© 2001 Cancer Research Campaign*

some role in explaining it for the 132 patients included in clinical trials.

Lead-time bias has been suggested as one possible explanation of improved survival in clinical trials: it cannot be totally ruled out in our study. The increase in the mean number of metastatic sites detected over time may reflect a real absence of lead-time bias or better detection of tumoral masses, but this better detection does not necessarily preclude the possibility that in fact tumoral mass might have diminished over time.

Only a few studies have analysed survival in population-based series of patients with SCLC. The US SEER population database showed a prolongation in the survival of SCLC patients: median survival time was 7.3 months in 1973–1974 and 9.3 months in 1993–1994 (Chute et al, 1999). The population database for the Southeastern Netherlands also showed improved survival for patients with SCLC from 1975–1979 (median survival: 5 months) to 1990–1992 (median survival: 9 months) (Janssen-Heijnen et al, 1998a).

Prognostic factors among patients with SCLC have been studied primarily among series of patients included in clinical trials. The prognostic role of age is quite controversial and there are some real difficulties in assessing its prognostic role. First, elderly patients are often excluded from clinical trials (Trumble et al, 1994). Second, the age cut-off varies significantly from study to study: 65 in a Belgian study (Humblet et al, 1987), 70 in a Danish study (Osterlind et al, 1986) and 75 in an English study (Souhami et al, 1984). Third, elderly patients are less likely to be treated aggressively than younger patients (Dajczman et al, 1996). It appears that when the elderly receive the same type of treatment as younger people do, age is not a prognostic factor (Sagman et al, 1991). The importance of appropriate treatment for the elderly is also supported by our finding that age was not significant after adjustment for treatment.

The prognostic value of sex is also controversial. In most North American series, prognosis is significantly more favourable among women (Davis et al, 1985; Johnson et al, 1988; Spiegelman et al, 1989; Wolf et al, 1991; Chute et al, 1997). Neither in our study nor in a French series of 1280 patients included in four consecutive trials did sex appear as a significant independent factor. The sex-ratio in that series was 9:1 (Lebeau et al, 1995). The low percentage of women with SCLC in France makes it difficult to ascertain whether sex has a prognostic role.

Performance status (PS) of patients is a major prognostic factor (Spiegelman et al, 1989; Albain et al, 1990; Rawson and Peto, 1990), especially for ED patients (Sagman et al, 1991). We could not study its prognostic role, however, because it was systematically recorded only for patients in clinical trials. For them, PS was an independent prognostic factor (data not shown).

Extent of disease is one of the strongest prognostic factors (Spiegelman et al, 1989; Albain et al, 1990; Lassen et al, 1995; Maestu et al, 1997), as our population-based series confirms. Some authors report the prognostic importance of the number of metastatic sites (Maurer and Pajak, 1981; Spiegelman et al, 1989; Sagman et al, 1991). We did not study this because this number depended on the number of imaging procedures.

LDH is now recognized as an important prognostic factor (Albain et al, 1990; Sagman et al, 1991; Maestu et al, 1997; Quoix et al, 2000). We could not assess it in our multivariate analysis because of the number of missing values.

Patients included in clinical trials survived longer than patients treated off-protocol. This reflects selection biases (Antman et al, 1985; Quoix et al, 1986). It is thus important to note that the results obtained with patients included in clinical trials cannot be extrapolated to the entire population of patients.

At the same time as survival improved over time in our series of patients, management of the disease also changed. The percentage of patients undergoing chemotherapy increased from 76.4% in 1981 to 91.7% in 1994. Janssen-Heijnen et al report that improved short-term survival in a population-based study was due to the increased use of chemotherapy from the late 1970s onwards (Janssen-Heijnen et al, 1998a). As in other countries, the combination of cisplatin and etoposide with or without other drug(s) became the standard chemotherapy in our region. In a meta-analysis of randomized trials of cisplatin-containing regimens versus regimens without cisplatin, the former was associated with a significant increase of survival of 2.6% at 6 months and 4.4% at 1 year (Pujol et al, 2000a). Paesmans et al showed that the administration of cisplatin and etoposide as components of a poly-chemotherapy regimen, separately or together, improved survival of SCLC patients (Paesmans et al, 1999). This review did not differentiate between cisplatin-etoposide only and cisplatin-etoposide associated with other drug(s). Combinations of cisplatin, etoposide plus other drug(s) yielded the best results in our study. This finding is consistent with the result of a French multi-centre study that compared doublet cisplatin plus etoposide to cisplatin plus etoposide plus cyclophosphamide plus epirubicin (Pujol, 2000b).

Mediastinal irradiation combined with chemotherapy is associated with better survival in patients with LD (Pignon et al, 1992; Warde and Payne, 1992). This was confirmed in our series. Moreover, during the second period there was a decrease of the median survival time in LD which may be related to a dramatic decrease of the percentage of LD patients treated with combination of chemotherapy plus mediastinal irradiation (29.8% versus at less 45% during the other periods). It is noteworthy that part of the dramatic decrease in the number of patients receiving mediastinal irradiation after the first period is due to the fact that this treatment was administered increasingly less often to ED patients.

The subset of patients in our study who were treated by surgery or received prophylactic cranial irradiation was too small to enable us to analyse their survival. The decrease of the number of patients receiving prophylactic cranial irradiation reflects probably the evolution of the management of the disease restricting this treatment to the patients responding completely to chemotherapy.

In conclusion, we observed in a French department population-based series of SCLC patients a 4.7-month improvement in median survival time, together with an 8% increase in the 2-year probability of survival over a 14-year period which is encouraging. The relative survival analysis confirmed the role of time in improvement of survival paralleling the evolution of the management of SCLC according to the 'state of the art'.

### ACKNOWLEDGMENTS

We are particularly indebted to Pr P Schaffer (Cancer Registry of Bas-Rhin) who gave us the list of all small-cell lung cancer patients diagnosed in the Bas-Rhin between 1981 and 1994.

We are also indebted to Dr E Achille (Clinique de l'Orangerie, Strasbourg), Pr JP Bergerat (Oncology, CHU Strasbourg), Dr P Charles (Clinique Ste Odile, Strasbourg), Dr MC Dickele (CHG, Haguenau), Dr B Gilet (CHG, Selestat), Dr B Orion (CHG,

*© 2001 Cancer Research Campaign* 

Saverne), Pr JL Schlienger (Internal Medicine, CHU Strasbourg), Pr S Schraub (Centre Paul Strauss, Strasbourg), Pr D Storck (Internal Medicine, CHU Strasbourg), Pr E Weitzenblum (Pneumology Hautepierre, CHU Strasbourg), and Pr JM Wihlm (Thoracic Surgery, CHU Strasbourg), who allowed us to review the patients' medical charts.

Supported in part by the 'Comité National contre les Maladies Respiratoires et la Tuberculose' (Paris, France).

### REFERENCES

Aisner J, Allort P, Bitran J et al (1983) Role of chemotherapy in small-cell lung cancer: a consensus report of the International Association for the Study of the Lung cancer workshop. *Cancer Treat Rep* **67**: 37–43

Albain KS, Crowley JJ, Leblanc M et al (1990) Determinants of improved outcome in small-cell lung cancer: an analysis of 2580 patients: Southwest Oncology Group Data Base. *J Clin Oncol* **8**: 1563–1574

Antman K, Amato D, Wood W et al (1985) Selection bias in clinical trials. *J Clin Oncol* **3**: 1142–1147

Armitage P (1971) *Statistical Methods in Medical Research*. Blackwell: Oxford

Black RJ, Bray F, Ferlay J et al (1997) Cancer incidence and mortality in the European union. Cancer Registry Data and Estimate of National Incidence for 1990. *Eur J Cancer* **33**: 1075–1107

BMDP (1981) Statistical Software. University of California Press: Berkeley, CA

Carter and Eggleston JC (1980), *Tumors of the lower respiratory tract*. Armed Forces Institute of Pathology

Chute JP, Venzon DJ, Hankins L et al (1997) Outcome of patients with small-cell lung cancer during 20 years of clinical research at the US National Cancer Institute. *Mayo Clin Proc* **72**: 901–912

Chute JP, Chen T, Feigal T et al (1999) Twenty years of phase III trials for patients with extensive-stage small-cell lung cancer: perceptible progress. *J Clin Oncol* **17**: 1794–1801

Dajczman E, Liyi F, Small D, Wolkove N and Kreisman H (1996) Treatment of small-cell lung carcinoma in the elderly. *Cancer* **77**: 2032–2038

Davis S, Wright PW, Schulman SF, Scholes D, Thorning D and Hammar S (1985) Long-term in small-cell carcinoma of the lung: a population experience. *J Clin Oncol* **3**: 80–91

Dearing MP, Steinberg SM, Phelps R et al (1990) Outcome of patients with small-cell lung cancer: effect of changes in staging procedures and imaging technology on prognostic factors over 14 years. *J Clin Oncol* **8**: 1042–1049

European Bureau for Action on Smoking Prevention (1990) Europe for smoking prevention. *Newsletter* **8**

Engeland A, Bjorge T, Haldorsen T and Tretlis (1998) Prognosis of patients with lung cancer diagnosed in Norway, 1954–93. *Cancer Causes Control* **9**: 57–65

Evans WK, Sheperd FA, Osoba D et al (1985) VP16 and cisplatin as first-line therapy for small-cell lung cancer. *J Clin Oncol* **3**: 1471–1447

Feinstein AR, Sosin DM and Well CK (1985) The Will Rogers Phenomenon: stage migration and new diagnostic techniques as a source of misleading statistics for survival in cancer. *N Engl J Med* **312**: 1604–1608

Fox W, Scadding JG and Medical Research Council (1973) Comparative trial of surgery and radiotherapy for primary treatment of small-cell or oat-celled carcinoma of the bronchus. Ten years follow-up. *Lancet* **2**: 63–65

Fry WA, Phillips JL et al (1999) Ten-Year Survey of Lung Cancer. Treatment and survival in hospitals in the United States. A National Cancer Data Base Report. *Cancer* **86**: 1867–1876

Fukuoka M, Furuse K, Saijo N et al (1991) Randomized trial of cyclophosphamide, doxorubicin, and vincristine versus cisplatin and etoposide versus alternation of these regimens in small-cell lung cancer. *J Natl Cancer Inst* **83**: 855–861

Giaccone G, Donadio M, Bonardi G et al (1988) Teniposide in the treatment of small-cell lung cancer: the influence of prior chemotherapy. *J Clin Oncol* **6**: 1264–1270

Green RA, Humphrey E, Close H et al (1969) Alkylating agents in bronchogenic carcinoma. *Am J Med* **46**: 516–525

Griffin CA, Lu C, Fishman EK et al (1984) The role of Computed Tomography of the chest in the management of small-cell lung cancer. *J Clin Oncol* **2**: 1359–1365

Hedelin G (1995) Relsurv A program for relative survival. Technical report of the department of epidemiology and public health, Faculty of medicine, Louis Pasteur University, Strasbourg, France

Humblet Y, Symann M, Bosly A et al (1987) Late intensification chemotherapy with autologous bone marrow transplantation in selected small-cell carcinoma of the lung: a randomized study. *J Clin Oncol* **5**: 1864–1873

Insee (1987) Estimation localisée de population

Janssen-Heijnen MLG, Nab HW, Van Reek J et al (1995) Striking changes in smoking behaviour and lung cancer incidence by histological type in the south-east Netherlands 1960–1991. *Eur J Cancer* **31A**: 949–952

Janssen-Heijnen MLG, Schipper RM, Klinkhamer PJJM et al (1998a) Improvement and plateau in survival of small-cell lung cancer since 1975: a population-based study. *Ann Oncol* **9**: 543–547

Janssen-Heijnen ML, Gatta G, Forman D, Capocaccia R and Coebergh JW (1998b) Variation in survival of patients with lung cancer in Europe, 1985–1989 Eurocare Working Group. *Eur J Cancer* **34**: 2191–2196

Johnson BE, Seth M et al (1988) Female patients with small-cell lung cancer live longer than male patients. *Am J Med* **85**: 194–196

Lassen U, Osterlind K, Hansen M et al (1995) Long-term survival in small-cell lung cancer: posttreatment characteristics in patients surviving 5 to 18 + Years. An analysis of 1714 consecutive patients. *J Clin Oncol* **13**: 1215–1220

Lebeau B, Chastang C, Schuller MP et al (1995) Chimiothérapie des cancers bronchiques à petites cellules. Importance pronostique d'une réponse complète (1280 patients). *La Presse Medicale* **24**: 217–221

Livingston RM, Moore TN, Heilbrun L et al (1978) Small cell carcinoma of the lung: combined chemotherapy and radiation. A Southwest Oncology Group Study. *Ann Intern Med* **88**: 194–199

Maestu I, Pastor M, Gomez-Codina J et al (1997) Pretreatment prognostic factors for survival in small-cell lung cancer: a new prognostic index and validation of three new prognostics indices on 341 patients. *Ann Oncol* **8**: 547–553

Maurer LH and Pajak TF (1981) Prognostic factors in small-cell carcinoma of the lung: a Cancer and Leukemia Group B Study. *Cancer Treat Rep* **65**: 767–774

Osterlind K and Andersen PK (1986) Prognostic factor in small-cell lung cancer: multivariate model based on 778 patients treated with chemotherapy with or without irradiation. *Cancer Res* **46**: 4189–4194

Paesmans M, Mascaux C, Berghmans T et al (1999) Etoposide and cisplatin merit their key role in chemotherapy for small-cell lung cancer: a meta-analysis with a methodology assessment, by the European Lung Cancer Working Party (ELCWP) *Am Soc Clin Oncol* **18**: 474a

Parkin DM, Ferlay J and Pisani P (1993) Estimate of the worldwide incidence of eighteen major cancers in 1985. *Intern J Cancer* **54**: 594–606

Perry MC, Eaton WL et al (1987) Chemotherapy with or without radiation therapy in limited small-cell carcinoma of the lung. *N Engl J Med* **316**: 912–918

Pignon JP, Arriagada R, Ihde D et al (1992) A meta-analysis of thoracic radiotherapy for small-cell lung cancer. *N Engl J Med* **327**: 1618–1624

Pujol JL, Carestia and Daures JP (2000a) Is there a case of cisplatin in the treatment of small-cell lung cancer? A meta-analysis of randomized trials of a cisplatin-containing regimen versus a regimen without this alkylating agent. *Br J Cancer* **83**: 8–15

Pujol JL (2000b) Doublet etoposide-cisplatin versus quadruplet cisplatin-cyclophosphamide-epirubicin-etoposide in extensive disease small-cell lung cancer. A FNCLCC Phase III multicentric study. *Proc ASCO* **19**: 484a

Quoix E, Finkelstein H, Wolkove N and Kreisman H (1986) Treatment of small-cell lung cancer on protocol: potential bias of results. *J Clin Oncol* **4**: 1314–1320

Quoix E, Purohit A, Faller-Beau M et al (2000) Comparative prognostic value of lactate dehydrogenase and neuron-specific enolase in small-cell lung cancer patients treated with platinum-based chemotherapy. *Lung Cancer* **30**: 127–134

Rawson NSB and Peto J (1990) An overview of prognostic factors in small-cell lung cancer. *Br J Cancer* **61**: 597–604

Roth BJ, Johnson DH, Einhorn LH et al (1992) Randomized study of cyclophosphamide, doxorubicine, and vincristine versus etoposide and cisplatin versus alternation of these two regimens in extensive small-cell lung cancer: a phase III trial of the Southeastern Cancer Study Group. *J Clin Oncol* **10**: 282–291

Sagman U, Maki E, Evans WK et al (1991) Small-cell carcinoma of the lung: derivation of a prognostic staging system. *J Clin Oncol* **9**: 1639–1649

Schwartz D (1996) *Méthodes statistiques à l'usage des Médecins et des Biologistes*. 4ème ed

Silverberg E (1998) *Cancer statistics 1988: a cancer Journal for Clinicians* **38**: 5–22

Souhami R, Geddes DM, Spiro SG et al (1984) Radiotherapy in small-cell of the lung treated with combination chemotherapy: a controlled trial. *BMJ* **288**: 1643–1656

Spiegelman D, Maurer LH, Ware LH et al (1989) Prognostic factors in small cell carcinoma of the lung: an analysis of 1521 patients. *J Clin Oncol* **7**: 344–354

Statxact 3 for Windows (1995) by Cyrus Metha and Nitin Patel. Founders of Cytel Software Corporation. Cambridge, MA 02139

© 2001 Cancer Research Campaign

Trumble EL, Carer CL, Cain D, Freindlin D, Ungecleider RS and Friedman MA (1994) Representation of older patients in cancer treatment trials. *Cancer* **74**: 2208–2214

Warde P and Payne D (1992) Does thoracic irradiation improve survival and local control in limited-stage small-cell carcinoma of the lung. *J Clin Oncol* **10**: 890–895

Wolf M, Holle R, Hans K et al (1991) Analysis of prognostic factors in 766 patients with small-cell lung cancer (SCLC): the role of sex as a predictor of survival. *Br J Cancer* **63**: 986–992

World Health Organization (1998) *Histological typing of tumors*. 3rd ed., Geneva

Zelen M (1973) Keynote Address on Biostatistics and Data Retrieval. *Cancer Chemother Rep* **4**: 31–42

*© 2001 Cancer Research Campaign*
